# The Importance of the Thyroid Nodule Location in Determining the Risk of Malignancy: A Retrospective Study

**DOI:** 10.7759/cureus.29421

**Published:** 2022-09-21

**Authors:** Raghd S Ageeli, Rwan A Mossery, Rahaf J Othathi, Ethar A Khawaji, Melia M Tarshi, Ghadah J Khormi, Sadeem M Bingasem, Rola A Khmees, Nada S Aburasain, Mohd Al Ghadeeb

**Affiliations:** 1 General Practice, Jazan University, Jazan, SAU; 2 Radiology, King Fahad Hospital, Hofuf, SAU

**Keywords:** malignancy, fine-needle aspiration, ultrasound, isthmus, thyroid nodule

## Abstract

Introduction

Thyroid nodules are very common. However, the majority of thyroid nodules are benign. Ultrasound is the first-line imaging investigation of choice for thyroid nodules. Certain sonographic features are associated with an increased risk of malignancy. Recent studies suggested that the location of the nodule may be associated with the malignancy risk. Hence, this study aims to investigate this association.

Methods

After obtaining approval from the ethics committee, we conducted a retrospective study that involved all patients who attended our hospital, and who underwent fine-needle aspiration cytology for the evaluation of suspicious thyroid nodules (TR3-5). Electronic medical records were used to obtain data about the ultrasound and cytology reports. A multivariable binary logistic regression analysis model was conducted to identify the independent factors significantly associated with malignant thyroid nodules.

Results

The study included 366 patients who underwent fine-needle aspiration cytology for suspicious nodules on thyroid ultrasound. In total, 52 (14.2%) nodules were found to be malignant on cytology. By far, the most common thyroid malignancy was papillary carcinoma. The multivariable analysis model revealed that women were 24% less likely to have malignant thyroid nodules compared with men. After adjusting for the age, gender, and Thyroid Imaging Reporting and Data System (TI-RADS) group, the nodules located within the isthmus were four times more likely to be malignant compared to those located in the right or left lobes.

Conclusions

The study demonstrates that the isthmus location of thyroid nodules is associated with a higher risk of malignancy. Physicians should have a lower threshold to biopsy such nodules. Further studies are needed to confirm this interesting finding.

## Introduction

Thyroid nodules are very common worldwide. They may come to clinical attention when they are noticed in clinical examinations or imaging studies. It is reported that the estimated prevalence of clinically palpable thyroid nodules is 3%-7% [1]. The prevalence of ultrasound-detected thyroid nodules is remarkably higher, ranging from 19% to 67% [2]. For example, in a large-scale study involving almost 100,000 ultrasound scans, one-third of the population had an ultrasound-detected thyroid nodule [3]. Thyroid nodules are more common in women and their prevalence increase with age [2].

The majority of thyroid nodules are benign. Nearly 5% of all thyroid nodules are malignant [4]. Certain risk factors have been identified to increase the risk of malignant thyroid nodules, such as age, gender, and history of radiation. The malignancy risk is greater in men and among patients aged less than 30 and greater than 60 years than those aged 30-60 years [5].

The major significance of thyroid nodules is related to the need to rule out malignancy. Ultrasound examination is used to provide the answer to the likelihood of malignancy in thyroid nodules. A number of features have been regarded to raise the concern for malignancy in thyroid nodules. However, there is no single sonographic feature that can be used to differentiate between benign and malignant nodules in isolation [6]. In order to minimize the number of unnecessary biopsies, several risk stratification systems have been developed to classify the nodules based on the risk of malignancy [7].

The Thyroid Imaging Reporting and Data System (TI-RADS) was developed by the American College of Radiology to standardize the reporting of thyroid ultrasound and to guide clinical decision-making [6]. The TI-RADS has five sonographic features, including composition, echogenicity, shape, margin, and echogenic foci. This system showed high sensitivity and specificity in the risk stratification of thyroid nodules [7].

The location and size of thyroid nodules are not part of the TI-RADS scoring system. Recent studies suggested that the location of thyroid nodules may be associated with an increased risk of malignancy [8]. Very few studies have focused on the association between thyroid isthmus nodules and malignancy. Therefore, this study aims to estimate the rate of malignancy in ultrasound-detected thyroid nodules and examine the association with the size and location of thyroid nodules.

## Materials and methods

Study design and settings

After obtaining approval from the ethics committee, we conducted a retrospective study to investigate the association between the location of thyroid nodules and malignancy. The study was conducted at the King Fahd Hospital in Al-Hofuf, one of the largest tertiary centers in the Eastern Province of Saudi Arabia. It has a bed capacity of 500 beds and serves as the primary public hospital in the Eastern Province.

Study population

The study involved all patients who attended the hospital between January 1, 2020, and December 31, 2021, and who underwent fine-needle aspiration cytology for the evaluation of suspicious thyroid nodules (TR3-5). Patients aged below 18 years were excluded.

Data collection

The data was collected by volunteering trainee residents with close supervision and guidance from the principal investigator. Structured data collection forms were developed to collect the demographic and sonographic characteristics. The demographic data included age, gender, and ethnicity. The radiological information system was utilized to obtain the radiologists’ reports for the thyroid ultrasound. The collected sonographic data of the thyroid nodules included the size, location, and TI-RADS category. All the data collection forms were double-checked for accuracy and completeness.

Ultrasound and cytology interpretations

In our hospital, all the ultrasound examinations are performed and reported by board-certified radiologists with experience of more than five years in general radiology practice. The radiologists apply the reporting system per the proposed guidelines of the American College of Radiology for thyroid nodules. Furthermore, the fine-needle aspiration cytology is also performed by radiologists using ultrasound guidance using 22-gauge needles.

Statistical analysis

The data were collected and compiled by Microsoft Excel 2016 (Microsoft, WA, USA). The analysis was made using Statistical Package for the Social Sciences (SPSS), version 26 (IBM Corp., Armonk, NY, USA). Figures were used to provide an illustrative summary of thyroid cancer types and thyroid nodule size and TI-RADS category according to their location. All the collected data were categorical. The data were presented as frequencies and percentages and compared using the chi-square test. A multivariable binary logistic regression analysis model was conducted to identify the independent factors significantly associated with malignant thyroid nodules. The independent factors in the model were selected based on the literature review and the bivariate analysis. Odds ratio (OR) were reported with the 95% confidence interval (CI). The significance level was defined as alpha = 0.05.

## Results

Participants characteristics

The study included 366 patients, including 45 men and 321 women, who underwent fine-needle aspiration cytology for suspicious nodules on thyroid ultrasound. Patients aged 36-50 years comprised the largest age group (60.7%), followed by those aged 18-35 years (26.5%). The vast majority (89.9%) of patients were Arabs (Table 1).

**Table 1:** Patients characteristics N: number of patients.

Variable		N	(%)
Age Group (years)	18–35	97	(26.5)
	36–50	222	(60.7)
	51–65	29	(7.9)
	>65	18	(4.9)
Gender	Male	45	(12.3)
	Female	321	(87.7)
Ethnicity	Arab	329	(89.9)
	Caucasian	18	(4.9)
	Asian	13	(3.6)
	Others	6	(1.6)

Table 2 illustrates the sonographic findings for the thyroid nodules. Most (70.2%) thyroid nodules were classified as the TR3 group. Only 14 (3.8%) nodules were classified as the TR5 group. Nearly two-thirds (65.8%) of nodules measured 2-4 cm in diameter, while only 38 (10.4%) nodules were larger than 4 cm. Regarding the location of the nodules, 190 (51.9%) and 115 (31.4%) were in the right and left lobe, respectively. In total, 61 nodules were within the isthmus.

**Table 2:** Sonographic findings of thyroid nodules N: Number of patients; TI-RADS: Thyroid Imaging, Reporting, and Data System.

Variable		N	(%)
TI-RADS Group	TR3	257	(70.2)
	TR4	95	(26.0)
	TR5	14	(3.8)
Nodule Size (cm)	1–2	87	(23.8)
	2–4	241	(65.8)
	>4	38	(10.4)
Nodule Location	Right Lobe	190	(51.9)
	Left Lobe	115	(31.4)
	Isthmus	61	(16.7)

Malignant thyroid nodules

**Figure 1:**
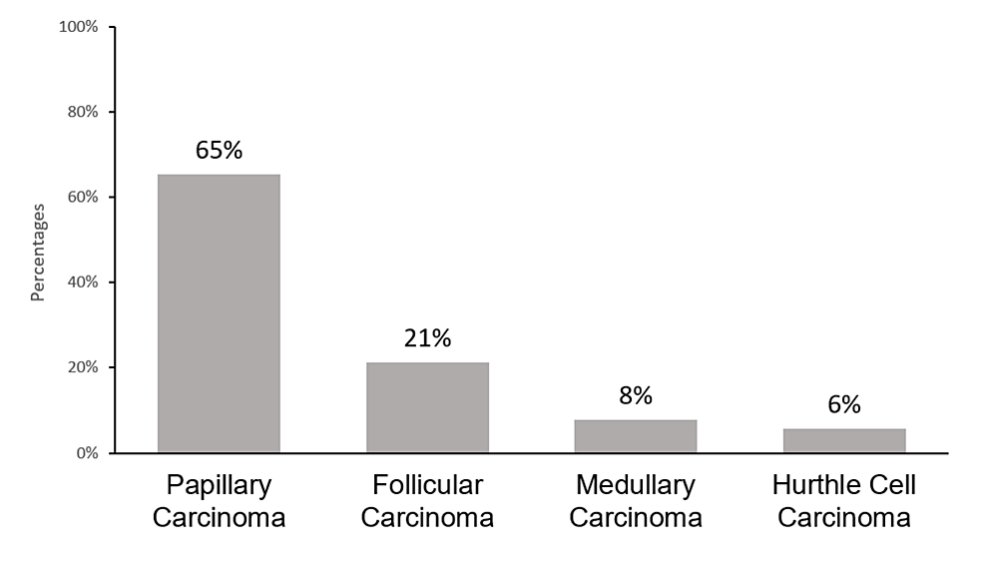
Types of thyroid cancers

In total, 52 (14.2%) nodules were found to be malignant on cytology. By far, the most common thyroid malignancy was papillary carcinoma, representing 65.4% of all thyroid malignancies, followed by follicular carcinoma seen in 21.2% of malignant nodules. There were four (7.7%) and three (5.8%) nodules having medullary thyroid carcinoma and Hurthle cell carcinoma, respectively (Figure 1).

Factors Associated with Malignant Thyroid Nodules

Table 3 summarizes the association of the demographic and sonographic findings with thyroid nodule malignancy. Regarding the demographic factors, the highest rate of malignancy was among patients aged 51-65 years, as nine (31.0%) thyroid nodules among this group were malignant (P = 0.01). While the majority of the study population were women (87.8%), men had a higher rate of thyroid nodule malignancy compared with women (28.9% vs 12.1%; P < 0.01).

**Table 3:** Demographic and sonographic findings associated with malignant nodules N: Number of patients; TI-RADS: Thyroid Imaging, Reporting, and Data System.

Variable		Malignant Nodules		P value
		N	(%)	
Age Group (years)	18–35	18	(18.6)	0.01
	36–50	23	(10.4)	
	51–65	9	(31.0)	
	>65	2	(11.1)	
Gender	Male	13	(28.9)	<0.01
	Female	39	(12.1)	
Ethnicity	Arab	50	(15.2)	0.02
	Caucasian	2	(11.1)	
	Asian	0	(0.0)	
	Others	0	(0.0)	
TI-RADS Group	TR3	17	(6.6)	<0.01
	TR4	21	(22.1)	
	TR5	12	(85.7)	
Nodule Size (cm)	1–2	14	(16.1)	0.56
	2–4	31	(12.9)	
	>4	7	(18.4)	
Nodule Location	Right Lobe	22	(11.6)	<0.01
	Left Lobe	13	(11.3)	
	Isthmus	17	(27.9)	

**Figure 2:**
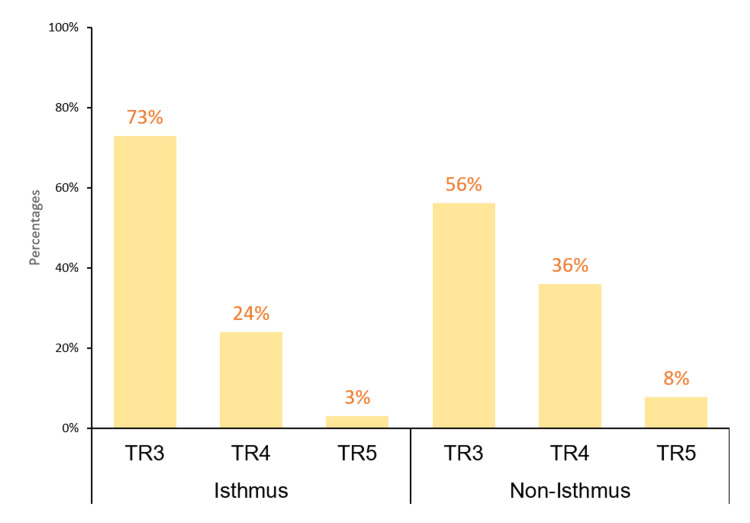
The TI-RADS category according to thyroid nodule location TI-RADS: Thyroid Imaging, Reporting, and Data System

**Figure 3:**
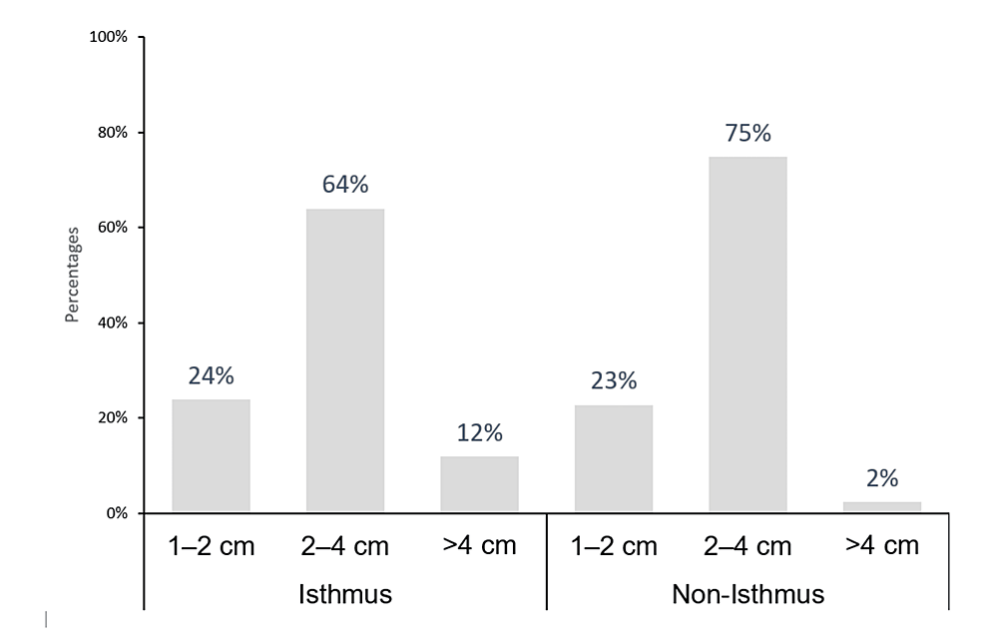
Thyroid nodule size according to location TI-RADS: Thyroid Imaging, Reporting, and Data System

The rate of malignancy was significantly associated with the TI-RADS group of the thyroid nodule (P < 0.01). In particular, the malignancy rate in TR3, TR4, and TR5 was 6.6%, 22.1%, and 85.7%, respectively. However, the size of the thyroid nodule was not significantly associated with the malignancy rate (P = 0.56). In contrast, the location of the thyroid nodule was significantly associated with malignancy. For instance, the nodules located in the isthmus had the highest rate (27.9%) of being malignant compared with those located in the right (11.6%) and left (11.3%) thyroid lobes. Notably, as illustrated in Figure 2 and Figure 3, the thyroid nodules located in the isthmus did not differ significantly in terms of their size and TI-RADS group compared with those seen in the right and left thyroid lobes (P > 0.05).

Multivariable Analysis of Factors Associated with Malignant Thyroid Nodules

Table 4 summarizes the independent demographic and sonographic features associated with malignancy among thyroid nodules. The multivariable analysis model revealed that women were 24% (0.16-0.87) less likely to have malignant thyroid nodules compared with men. Furthermore, nodules in the TR4 and TR5 were 5- and 20-times more likely to be malignant compared to those classified in the TR3 group. Additionally, the nodules located within the isthmus were four times more likely to be malignant compared to those located in the right or left lobes (OR = 4.1; 95% CI: 2.1-12.6).

**Table 4:** Multivariable analysis of factors associated with malignant nodules TI-RADS: Thyroid Imaging, Reporting, and Data System; OR: odds ratio; CI: confidence interval.

Variable		Unadjusted OR (95% CI)	Adjusted OR (95% CI)	P value
Age Group (years)	18–35	Reference Category		
	36–50	0.51 (0.26–0.99)	0.88 (0.57–2.64)	0.76
	51–65	1.98 (0.77–5.05)	3.21 (0.45–10.88)	0.15
	>65	0.55 (0.12–2.60)	0.48 (0.33–9.42)	0.40
Gender	Male	Reference Category		
	Female	0.34 (0.17–0.70)	0.24 (0.16–0.87)	<0.01
TI-RADS Group	TR3	Reference Category		
	TR4	4.01 (2.01–7.99)	5.65 (3.87–15.72)	<0.01
	TR5	84.71 (17.52–409.48)	20.41 (10.11–36.78)	<0.01
Nodule Location	Right/Left Lobe	Reference Category		
	Isthmus	2.98 (1.54–5.77)	4.07 (2.14–12.64)	<0.01

## Discussion

The study aimed to investigate the association between the location of thyroid nodules and the risk of malignancy. It revealed that even after adjusting for the TI-RADS group, the isthmus location of thyroid nodules was an independent factor associated with a higher risk of malignancy. This interesting finding is consistent with a number of recent studies [8,9]. For example, in a retrospective study involving over 3,000 patients with thyroid nodules, it was found that the isthmus location had a nearly 2.5-times higher risk of being malignant compared with lobar nodules [9]. Similarly, Pastorello et al. showed that although only 10% of thyroid malignancies developed in the isthmus, the malignancy rate was the highest in the nodules of the isthmus [8].

It is also interesting to note that malignant thyroid nodules of the isthmus have a higher tendency to develop a persistent disease after treatment and have shorter disease-free survival compared to thyroid malignancies in the right or left lobes [10]. Furthermore, a retrospective study involving 190 patients with papillary thyroid cancer found that the cancers located in the isthmus had a higher rate of extra-thyroid extension and nodal metastasis [11].

The increased malignancy risk among thyroid nodules of the isthmus remains unclear. It was suggested that the embryological nature and cellular composition of the isthmus are different than those of the right and left thyroid lobes [12]. It has also been suggested that the superficial location of the isthmus nodules leads to an earlier diagnosis as they are easily accessible for biopsy sampling [8]. A prior study revealed that the nodules of the isthmus tend to have a smaller size [9]. However, our findings showed that the nodules in the isthmus have similar characteristics in terms of size and TI-RADS category compared with those nodules developing in the right and left lobes. The size of the nodule showed no significant association with the risk of malignancy.

The current study has some limitations. First, it is a single-center study; however, it has a sufficient sample size. Second, the study did not investigate the levels of thyroid-stimulating hormones among the study population, which may have a prognostic influence on thyroid nodules [13]. Third, the retrospective nature of the study is another limitation, as it highly relied on the accuracy of the electronic medical records which may have some missing or inaccurate data.

## Conclusions

This study along with other similar studies in the literature demonstrates that the location of thyroid nodules in the isthmus carries a higher risk of malignancy. This may suggest that physicians should have a lower threshold to biopsy the nodules in the thyroid isthmus. Further studies with a larger population are needed to establish this finding as the location of the nodule may be a potential component of future risk-stratification systems.
